# Sex inequality in early initiation of breastfeeding in 24 sub-Saharan African countries: A multi-country analysis of Demographic and Health Surveys

**DOI:** 10.1371/journal.pone.0267703

**Published:** 2022-05-19

**Authors:** Obasanjo Afolabi Bolarinwa, Bright Opoku Ahinkorah, Francis Arthur-Holmes, Richard Gyan Aboagye, Edward Kwabena Ameyaw, Eugene Budu, Abdul-Aziz Seidu, Uzairue Leonard Ighodalo, Sanni Yaya

**Affiliations:** 1 Department of Public Health Medicine, School of Nursing and Public Health, University of KwaZulu-Natal, Durban, South Africa; 2 School of Public Health, Faculty of Health, University of Technology Sydney, Sydney, NSW, Australia; 3 Department of Sociology and Social Policy, Lingnan University, Tuen Mun, Hong Kong; 4 Department of Family and Community Health, School of Public Health, University of Health and Allied Sciences, Hohoe, Ghana; 5 Department of Population and Health, University of Cape Coast, Cape Coast, Ghana; 6 College of Public Health, Medical and Veterinary Sciences, James Cook University, Townsville, QLD, Australia; 7 Department of Estate Management, Takoradi Technical University, Takoradi, Ghana; 8 Centre for Gender and Advocacy, Takoradi Technical University, Takoradi, Ghana; 9 Department of Medical Laboratory Science, Faculty of Basic Medical Science, Federal University, Oye Ekiti, Ekiti State, Nigeria; 10 School of International Development and Global Studies, Faculty of Social Sciences, University of Ottawa, 120 University Private, Ottawa, ON, Canada; 11 The George Institute for Global Health, Imperial College London, London, United Kingdom; University of Salamanca, SPAIN

## Abstract

**Background:**

The Sustainable Development Goal (SDG) 3 aims at reducing neonatal and under-5 mortality to below 12 per 1000 and 25 per 1000 live births, respectively, globally by 2030. Studies have found that initiation of breastfeeding within one hour of birth and continuous breastfeeding for over 12 months can positively impact neonatal and infant health. However, there is evidence that the sex of a child may influence the breastfeeding practices of a mother. Thus, we examined sex inequality in early breastfeeding initiation in sub-Saharan Africa.

**Materials and methods:**

Data from Demographic and Health Surveys conducted in 24 sub-Saharan African countries between January 2010 and December 2019 were pooled and analysed. A total of 137,677 women of reproductive age (15–49 years) were considered in this study. Bivariate and multivariable regression analyses were performed, and the results were presented using crude odds ratio (cOR) and adjusted odds ratio (aOR) with statistical significance at a p-value less than 0.05.

**Results:**

The highest inequality in early initiation of breastfeeding was reported in Togo with a difference of 5.21% between the female and male children, while the lowest inequality was reported in Guinea with 0.48% difference between the female and male children. A higher odds of breastfeeding within 1 hour was observed among female children [cOR = 1.05; 95%(CI = 1.02–1.09)] compared to male children, and this persisted after controlling for the confounders included in this study [aOR = 1.05; 95%(CI = 1.02–1.08)].

**Conclusion:**

We found higher odds for early breastfeeding initiation of female children compared to male children in sub-Saharan Africa. To reduce breastfeeding initiation inequalities, programmes that educate and encourage early initiation of breastfeeding irrespective of the child sex should be promoted among mothers.

## Background

The World Health Organization (WHO), in the implementation guideline released in 2017, recommends that breastfeeding begins within the first hour of birth [1–3]. However, a study conducted in 2015 reported that more than half of mothers delay breastfeeding initiation [4]. One of the targets of the third Sustainable Development Goal (SDG) is to reduce neonatal and under-5 mortality to below 12 per 1000 and 25 per 1000 live births, respectively by 2030 [5]. However, in 2019, WHO and World Bank reports showed that countries in sub-Saharan Africa had a neonatal mortality rate of 27 deaths per 1,000 live births [6] and 76 deaths per 1,000 live births [7].

Early initiation of breastfeeding (EIBF) defined as the initiation of breastfeeding within the first hour (1 hour) of birth [4] is considered an “essential newborn care” [8]. This is because EIBF is beneficial to both the newborn and the mother [9]. For the newborn, EIBF results in colostrum production, which provides vital nutrients and additional protective benefits for the baby (2), and for the mother, it triggers the release of hormones that help the mother’s uterus contract and prevent postpartum hemorrhage [10].

Studies have found that initiation of breastfeeding within one hour of birth and continuous breastfeeding for over 12-months have positive impact in reducing neonatal and infant mortality [11–15]. For instance, a systematic review concluded that over 40% reduction in neonatal and child mortality rate was reported for early breastfeeding initiation within 24 hours of birth [16, 17]. EIBF also provides thermal care for the newborn through skin-to-skin contact with the mother [18, 19]. Despite these benefits, disparities in EIBF practice have been reported in some regions. For instance, South and Southeast Asia had EIBF ranges from 20% in Pakistan to 76% in Timor-Leste [20]. This same level of disparity was reported in some sub-Saharan African countries as well, where 85% of babies in Burundi began breastfeeding immediately and just 23% in Chad [20]. Apart from the country-level differences in prevalence of EIBF, there is evidence that the sex of a child also plays a role in the timing of EIBF. A study conducted in Bangladesh by Sen, Mallick [9] reported that female children had higher odds of EIBF compared to male children. A study in West Africa found that EIBF was significantly higher with female births than male births (38). On the contrary, Seidu et al. [21] found that EIBF was higher in male children (51.5%) compared to females (48.5%) in Ghana.

Despite evidence that the sex of a child plays a role in the timing of EIBF, comprehensive evidence on the sex inequality in EIBF is scanty in sub-Saharan Africa (SSA). Therefore, there is a need to examine the sex inequality in EIBF in SSA using the most recent available Demographic and Health Survey (DHS) datasets. Hence, this study examined the sex inequality in EIBF in 24 countries in SSA using data from DHS. Evaluating sex inequality in EIBF in SSA can provide findings to assist policymakers and health professionals in implementing appropriate interventions to change social norms and mothers’ behaviours that lead to delayed initiation of breastfeeding. The findings will also help in knowing which particular child sex is mostly breastfed after 1 hour so that healthcare professionals can provide support and direction to prospective mothers on breastfeeding their newborn babies as well as the consequences of delays in breastfeeding initiation.

## Materials and methods

### Study design and data source

This study involved a cross-sectional analysis of DHS data from 24 sub-Saharan African countries. DHS is a nationally representative study conducted in over eighty-five low-and-middle-income countries (LMICs). The survey employed a questionnaire to collect data from respondents on several health indicators such as maternal and child health, men’s health, family planning, fertility, gender-based violence, substance use, Human Immunodeficiency Virus/Acquired Immunodeficiency Syndrome (HIV/AIDS), and nutrition [22]. Respondents for the survey were sampled using a two-stage cluster random sampling technique. The study by Aliaga and Ruilin [23] highlights the detailed sampling processes used in the DHS. The present study sample was drawn from the birth recode’s files from all the countries used. A total of 137,677 women aged 15–49 who had complete cases of the studied variables on questions about breastfeeding of the last child they had 5 years preceding the survey were included in the final analysis. Other respondents with incomplete information about the study of interest were dropped from the analysis. We relied on the Strengthening the Reporting of Observational Studies in Epidemiology (STROBE) statement in drafting the manuscript [24]. Sample size distribution by country and survey year are presented in Table 1. The datasets for the DHS can be accessed freely at https://dhsprogram.com/data/available-datasets.cfm.

**Table 1:** Sample size distribution by country and survey year.

Survey countries	Survey year	Weighted sample	Percentage (%)
**Central Africa**			
1. Angola	2016	5130	3.73
2. Congo-DR	2014	10770	7.82
3. Republic of Congo	2012	5476	3.98
4. Cameroon	2019	3605	2.62
5. Gabon	2012	3339	2.43
6. Rwanda	2015	5985	4.35
7. Chad	2015	10611	7.71
**West Africa**			
8. Burkina Faso	2010	10299	7.48
9. Benin	2018	5312	3.86
10. Côte d’Ivoire	2012	4915	3.57
11. Gambia	2013	5182	3.76
12. Guinea	2018	2800	2.03
13. Liberia	2013	4652	3.38
14. Nigeria	2018	12562	9.12
15. Sierra Leone	2013	8233	5.98
16. Senegal	2018	2393	1.74
17. Togo	2014	4723	3.43
18. Ghana	2014	4080	2.96
**East Africa**			
19. Burundi	2017	5355	3.89
20. Ethiopia	2016	4169	3.03
21. Uganda	2016	4840	3.52
**Southern Africa**			
22. Comoros	2012	1782	1.29
23. Mozambique	2011	7641	5.55
24. Zimbabwe	2018	3823	2.78

### Study variables

#### Outcome variable

The outcome variable in this study was EIBF. EIBF is defined as the initiation of breastfeeding within the first hour (1 hour) of birth [4, 25]. From the DHS, the respondents were asked when they started to breastfeed their newborn after birth. The responses were documented in “immediately,” “hours,” and “days”. The responses were further re-categorized into EIBF (within 1 hour of birth) and late breastfeeding initiation (More than 1 hour). Similar coding has been used in several studies [9, 26, 27].

#### Key explanatory variable

The main explanatory variable was the child’s sex. The categorization of this variable was “Male” and “Female”. A study by Sen, Mallick [9] used the same categorization to assess inequalities in EIBF.

#### Covariates

A total of thirteen (13) covariates were studied. These variables consist of maternal age, age at first birth, assistance at birth, place of residence, maternal educational level, partner educational level, parity, wanted last child, place of delivery, delivery by cesarean section, antenatal care (ANC) visit during pregnancy, wealth index, and media exposure. These variables were not determined a priori; instead, based on parsimony and significant association with EIBF [9, 28–30]. Except for the place of residence and wealth index where the existing DHS coding was used, the remaining covariates were recoded. The other covariates and their recoding include maternal age (15–24, 25–34, and 35 and above); age at first birth (below 20 years and 20 years and above); assistance at birth (unskilled and skilled); maternal educational level (no education, primary, and secondary or higher); partner’s educational level (no education, primary, and secondary or higher); parity (1–3 and 4 and above); wanted last child (wanted and unwanted); place of delivery (home and health facility); delivery by cesarean section (No/Yes); ANC visits during pregnancy (none, less than 4, and 4 or more); and media exposure (No/Yes). Exposure to radio, television, newspaper/magazine was coded as media exposure. Media exposure was derived from these three variables using panel analysis. “Yes” means exposure to mass media while “No” means no exposure to mass media.

### Statistical analyses

Data extraction, cleaning, recoding, and analyses were carried out using Stata software version 16.0 (Stata Corporation, College Station, TX, USA). Bar chart was used to show the sex disparities in EIBF by country. Next, the Pearson chi-square test was conducted to determine the relationship between the mother and child’s characteristics and EIBF. After this, two regression models were built to determine the associations between sex of the child, the covariates, and EIBF. Specifically, the first model (bivariate regression) examined the independent associations between sex of the child, each covariate, and EIBF. The second model (multivariable regression) was used to determine the association between a child’s sex and EIBF while controlling for the covariates. The results of the regression analyses were presented in a tabular form using crude odds ratio (cOR) and adjusted odds ratio (aOR) with their respective 95% confidence interval (CIs). Finally, the crude and adjusted results on the association between sex of the child and EIBF were disaggregated by country. Statistical significance at p-value less than 0.05. All the frequency distributions were weighted using the DHS recommended weight of v005/1,000,000 to avoid oversampling and non-response error. The survey Stata command (svy) was used to adjust to the complex sampling structure of the DHS data in the chi-square and regression analyses. The multicollinearity test, which used the variance inflation factor (VIF), revealed no evidence of collinearity amongst the independent variable and covariates.

### Ethical approval

Since the authors of this manuscript did not collect the data, we sought permission from the MEASURE DHS website and access to the data was provided after our intent for the request was assessed and approved on the 10th of January 2021. The DHS surveys are ethically accepted by the ORC Macro Inc. Ethics Committee and the Ethics Boards of partner organizations in different countries, such as the Ministries of Health. The women who were interviewed gave either written or verbal consent during each of the surveys.

## Results

**Fig 1:**
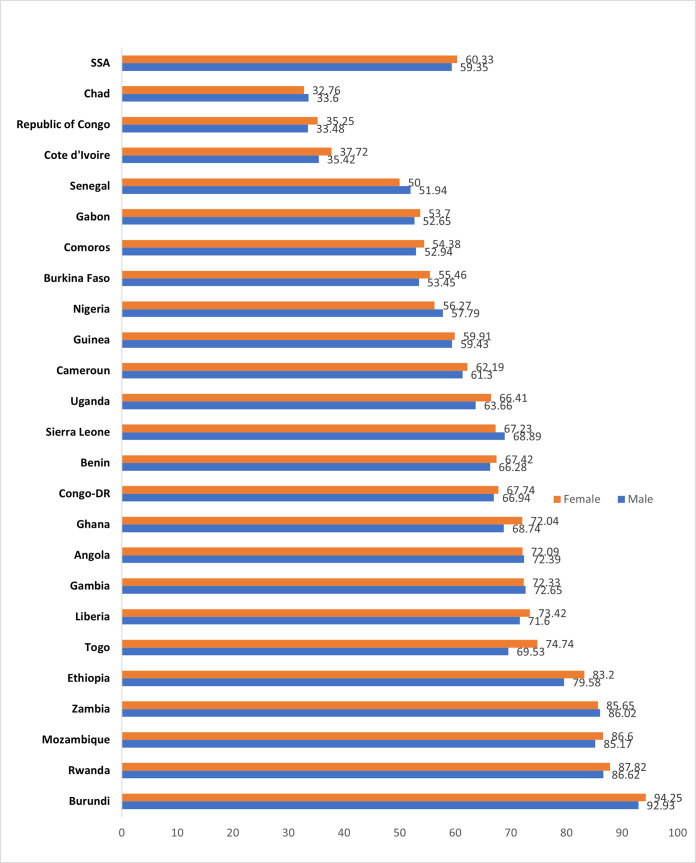
Sex inequalities in early breastfeeding initiation in SSA.

Fig 1 shows the sex inequality in early breastfeeding in SSA.

It showed that male children experienced inequality in early breastfeeding after birth compared to their female counterparts. The highest inequality was reported in Togo with a difference of 5.21% between the female and male child, while the lowest inequality was reported in Guinea with a 0.48% difference between the female and male child.

Table 2 shows breastfeeding initiation distribution among women in SSA by the key explanatory variable and covariates. The key explanatory variable shows inequality in EIBF with 36.88% of male children being breastfed late (more than 1 hour) after birth. Furthermore, a high prevalence in late breastfeeding initiation (More than 1hour) was found among women aged 15–24 (38.14%), those who had their first child below age 20 (37.91%), women who had assisted birth by unskilled professionals (38.60%), those residing in the urban (38.17%), women whose partner has no education (38.28%), those who had birth home delivery (43.15%), women who had a delivery through cesarean section (59.00%), those with no visit to antenatal during pregnancy (43.33%) and women who were not exposed to media (37.09%). Both key explanatory variable and covariates p-values were either significant at 0.001, 0.01, or 0.05 except wanted last-child variable that p-value was more than 0.05.

**Table 2:** Distribution of breastfeeding initiation among women by key independent variable and covariates (N = 137,677).

Variables	Frequency (n)	Percentage (%)	Breastfeeding initiation		p-value*
Key independent			More than 1hour	1 hour & below	
**Sex of child**					p<0.001
Male	69,555	50.52	36.88	63.12	
Female	68,122	49.48	35.66	64.34	
Covariates					
**Age**					p<0.001
15–24	42,959	31.20	38.14	61.86	
25–34	63,688	46.26	35.59	64.41	
35 & above	31,030	22.54	35.10	64.90	
**Age at first birth**					p<0.001
20 & above	55,429	40.26	33.85	66.15	
Below 20	82,249	59.74	37.91	62.09	
**Assistant at birth**					p<0.001
Unskilled	106,947	77.68	38.60	61.40	
Skilled	30,730	22.32	28.20	71.80	
**Place of residence**					p<0.001
Urban	46,496	33.77	38.17	61.83	
Rural	91,182	66.23	35.31	64.69	
**Mother’s Educational level**					p<0.001
No Education	59,398	43.14	38.23	61.77	
Primary	42,783	30.35	31.78	68.22	
Secondary/Higher	36,496	26.51	38.24	61.76	
**Partner’s Educational level**					p<0.001
No Education	61,905	44.96	38.28	61.72	
Primary	31,251	22.70	29.56	70.44	
Secondary/Higher	44,522	32.34	38.20	61.80	
**Parity**					p<0.01
1–3 children	74,829	54.35	36.67	63.33	
4 and above	62,848	45.65	35.80	64.20	
**Wanted last-child**					0.93
Wanted	103,387	75.09	36.29	63.71	
Unwanted	34,290	24.91	36.24	63.76	
**Place of delivery**					p<0.001
Home delivery	44,439	32.28	43.15	56.85	
Health facility delivery	93,228	67.72	33.00	67.00	
**Delivery by Caesarean**					p<0.001
No	130,975	95.13	35.11	64.89	
Yes	6,702	4.87	59.00	41.00	
**ANC visit during pregnancy**					p<0.001
No visit	17,289	12.56	43.33	56.67	
Less than 4 visits	45,311	32.92	35.47	64.53	
4 or more visits	75,057	54.52	35.13	64.87	
**Wealth index**					p<0.05
Poorest	29,888	21.71	36.94	63.06	
Poorer	29,790	21.64	36.61	63.39	
Middle	28,139	20.44	35.12	64.88	
Richer	26,497	19.25	35.61	64.39	
Richest	23,363	16.97	37.15	62.85	
**Media exposure**					p<0.01
No	50,257	36.50	37.09	62.91	
Yes	87,420	63.50	35.81	64.19	

Weighted

*P-values obtained from Pearson chi-square test

### Association between child sex and early initiation of breastfeeding among women in sub- Saharan Africa

The results of the association between sex of the child and EIBF are presented in Table 3. The adjusted model (Model II) indicated that female children [(aOR = 1.05, 95% CI = (1.02–1.08)] had higher odds of being breastfed within an hour after birth compared to the male child. The results in Model II further showed that women of 35 years and above [(aOR = 1.13, 95% CI = (1.06–1.19)], those who had skill assistant at birth [(aOR = 1.18, 95% CI = (1.11–1.26)], women whose partner has primary education [(aOR = 1.22, 95% CI = (1.17–1.28)], those who delivered their child at the health facilities [(aOR = 1.42, 95% CI = (1.35–1.50)], and those within the richest wealth index [(aOR = 1.13 95% CI = (1.04–1.23)] had higher odds of breastfeeding their children within an hour after birth while those who didn’t want the last child [(aOR = 0.93, 95% CI = (0.90–0.97)], and women who had their delivery by caesarean [(aOR = 0.30, 95% CI = (0.27–0.32)], had reduced odds of initiating breastfeeding within an hour after birth compared to mothers whose partners were without education, those who had non-caesarean delivery, and those who are unexposed to mass media.

**Table 3:** Bivariate and multivariable models showing the effect of sex of the child, selected covariates, and early initiation of breastfeeding among women in sub-Saharan African countries.

Variable	Unadjusted (Model I)			Adjusted (Model II)		
**Key Independent**	cOR	95% CI	P-value	aOR	95% CI	P-value
**Sex**						
Male	Reference (1.0)			Reference (1.0)		
Female	1.05***	1.02–1.09	0.000	1.05**	1.02–1.08	0.002
**Covariates**						
**Age**						
15–24	Reference (1.0)			Reference (1.0)		
25–34	1.12***	1.08–1.15	0.000	1.09***	1.04–1.14	0.000
35 & above	1.14***	1.10–1.18	0.000	1.13***	1.07–1.19	0.000
**Age at first birth**						
20 & above	Reference (1.0)			Reference (1.0)		
Below 20	0.84***	0.81–0.87	0.000	0.91***	0.88–0.95	0.000
**Assistant at birth**						
Unskilled	Reference (1.0)			Reference (1.0)		
Skilled	1.60***	1.52–1.69	0.000	1.18***	1.11–1.26	0.000
**Place of residence**						
Urban	Reference (1.0)			Reference (1.0)		
Rural	1.13***	1.07–1.20	0.000	1.16***	1.08–1.24	0.000
**Mother’s Educational level**						
No Education	Reference (1.0)			Reference (1.0)		
Primary	1.33***	1.27–1.39	0.000	1.14***	1.09–1.20	0.000
Secondary/Higher	1.00	0.95–1.05	0.994	0.97	0.92–1.02	0.223
**Partner’s Educational level**						
No Education	Reference (1.0)			Reference (1.0)		
Primary	1.48***	1.41–1.55	0.000	1.22***	1.17–1.28	0.000
Secondary/Higher	1.00	0.96–1.05	0.878	0.95*	0.91–0.99	0.026
**Parity**						
1–3 children	Reference (1.0)			Reference (1.0)		
4 and above	1.04**	1.01–1.07	0.012	1.00	0.96–1.05	0.872
**Wanted last-child**						
Wanted	Reference (1.0)			Reference (1.0)		
Unwanted	1.00	0.96–1.04	0.926	0.93**	0.90–0.97	0.001
**Place of delivery**						
Home delivery	Reference (1.0)			Reference (1.0)		
Health facility delivery	1.54***	1.47–1.62	0.000	1.42***	1.35–1.50	0.000
**Delivery by Caesarean**						
No	Reference (1.0)			Reference (1.0)		
Yes	0.38***	0.35–0.41	0.000	0.30***	0.27–0.32	0.000
**ANC visit during pregnancy**						
No visit	Reference (1.0)			Reference (1.0)		
Less than 4 visits	1.39***	1.30–1.49	0.000	0.99	0.92–1.06	0.792
4 or more visits	1.41***	1.32–1.51	0.000	1.06	0.99–1.13	0.084
**Wealth index**						
Poorest	Reference (1.0)			Reference (1.0)		
Poorer	1.01	0.97–1.06	0.557	1.00	0.95–1.05	0.901
Middle	1.08**	1.02–1.15	0.007	1.09**	1.02–1.16	0.005
Richer	1.06*	0.99–1.13	0.082	1.10**	1.02–1.19	0.009
Richest	0.99	0.92–1.06	0.798	1.13	1.04–1.23	0.004
**Media exposure**						
No	Reference (1.0)			Reference (1.0)		
Yes	1.06**	1.01–1.10	0.012	0.98	0.94–1.03	0.462
Survey’s year						
2010	Reference (1.0)			Reference (1.0)		
2011	0.36***	0.30–0.42	0.000	1.05	0.91–1.22	0.480
2012	0.16***	0.13–0.18	0.000	0.56***	0.49–0.63	0.000
2013	0.43***	0.36–0.50	0.000	1.05	1.02–1.18	0.462
2014	0.33***	0.28–0.39	0.000	1.51	0.32–1.72	0.000
2015	0.21***	0.18–0.25	0.000	0.95	0.83–1.08	0.429
2016	1.08	0.88–1.31	0.462	1.08	0.94–1.24	0.281
2017	0.78*	0.65–0.94	0.011	6.51***	5.53–7.66	0.000
2018	0.30***	0.25–0.35	0.000	0.91	0.80–1.05	0.194
2019	1.96	0.88–4.35	0.100	1.05	0.90–1.23	0.510

Model 1: unadjusted model examining the independent association of child’s sex and breastfeeding initiation; Model 2: adjusted for the covariates; AOR is the adjusted odds ratio, COR is the unadjusted odds ratio; Exponentiated coefficients; 95% CI is the 95% confidence intervals.

*p <0.05

**p <0.01

***p <0.001.

To show the variation in sex inequality in breastfeeding initiation between countries, two models were fitted to examine the association between a child’s sex and EIBF, with the results presented in Table 4. Model I was a crude model with no covariates, while Model II adjusted for the covariates. In Model I, a statistically significant effect of a child’s sex and breastfeeding initiation was found in Ghana, Burkina Faso, Togo, Burundi, Ethiopia, and Uganda, where the odds of EIBF was higher among women with female children compared to male children. In the adjusted Model (Model II), the odds of EIBF were high among women with female children compared to male children in Bukina Faso [aOR = 1.09; 95%(CI = 1.01–1.180)], Togo [aOR = 1.26; 95%(CI = 1.11–1.44)], Burundi [aOR = 1.31; 95%(CI = 1.03–1.66)], and Ethiopia [aOR = 1.27; 95%(CI = 1.08–1.50)].

**Table 4:** Bivariate and multivariable models showing the effect of sex of child and early initiation of breastfeeding among women in sub-Saharan Africa countries.

Survey countries	Model I COR [95% CI)	P-value	Model II AOR [95% CI]	P-value
**Central Africa**				
Angola	0.98 [0.88–1.11]	0.807	1.00 [0.88–1.12]	0.923
Congo-DR	1.04 [0.95–1.22]	0.373	1.02 [0.94–1.10]	0.654
Republic of Congo	1.08 [0.97–1.20]	0.142	1.07 [0.96–1.19]	0.213
Cameroon	1.04 [0.91–1.19]	0.590	1.04 [0.91–1.20]	0.576
Gabon	1.09 [0.96–1.23]	0.203	1.06 [0.93–1.21]	0.346
Rwanda	1.11 [0.96–1.30]	0.167	1.05 [0.89–1.24]	0.557
Chad	0.96 [0.89–1.04]	0.361	0.96 [0.88–1.05]	0.417
**West Africa**				
Burkina Faso	1.08* [1.00–1.17]	0.042	1.09* [1.01–1.18]	0.025
Benin	1.05 [0.94–1.18]	0.376	1.03 [0.91–1.15]	0.646
Côte d’Ivore	1.10 [0.99–1.24]	0.088	1.12 [1.00–1.26]	0.053
Gambia	0.98 [0.87–1.11]	0.796	1.00 [0.88–1.13]	0.928
Guinea	1.02 [0.88–1.19]	0.794	1.04 [0.89–1.21]	0.594
Liberia	1.10 [0.97–1.24]	0.142	1.07 [0.95–1.21]	0.274
Nigeria	0.94 [0.88–1.01]	0.088	0.94 [0.87–1.01]	0.072
Sierra Leone	0.93 [0.84–1.02]	0.109	0.92 [0.84–1.01]	0.077
Senegal	0.93 [0.79–1.08]	0.319	0.91 [0.78–1.07]	0.237
Togo	1.30*** [1.14–1.47]	0.000	1.26*** [1.11–1.44]	0.000
Ghana	1.17* [1.03–1.34]	0.019	1.14 [0.99–1.30]	0.065
**East Africa**				
Burundi	1.25* [1.00–1.56]	0.052	1.31* [1.03–1.66]	0.030
Ethiopia	1.27** [1.08–1.49]	0.003	1.27** [1.08–1.50]	0.004
Uganda	1.13* [1.00–1.27]	0.046	1.13* [1.00–1.28]	0.043
**Southern Africa**				
Comoros	1.06 [0.88–1.28]	0.550	1.10 [0.91–1.34]	
Mozambique	1.13 [0.99–1.28]	0.077	1.13 [0.99–1.29]	0.069
Zimbabwe	0.97 [0.81–1.16]	0.742	0.97 [0.80–1.18]	0.786

Model 1: unadjusted model examining the independent association of child’s sex and breastfeeding initiation; Model 2: adjusted for socio-demographic and other related factors (age, age at first birth, assistant at birth, place of residence, maternal educational level, partner’s educational level, parity, wanted last-child, place of delivery, delivery by cesarean, ANC visit during pregnancy, wealth index, and media); AOR is the adjusted odds ratio, COR is the unadjusted odds ratio.

*p <0.05

**p <0.01

***p <0.001.

## Discussion

Examining childhood inequality in early breastfeeding initiation is crucial to implementing specific health interventions to promote babies’ wellbeing. This study assessed children’s sex inequality in EIBF in SSA as studies have failed to investigate these important dynamics in the sub-region. Delayed breastfeeding initiation increases the risk of neonatal mortality [31, 32]. EIBF has been found to have benefits, including reducing the use of prolateral feeds that have a high risk of contamination and limited protection against respiratory infections [33, 34]. The study found inequality in the EIBF, with 36.88% of male children being breastfed late (more than 1 hour) after birth. This finding suggests male children who are breastfed late could have higher risks of neonatal death than female children, as indicated in previous studies [31, 33, 34]. There might be several reasons mothers breastfeed male children late after giving birth compared to female children.

Our study found a higher odds of EIBF among female children in six sub-Saharan African countries. These are Togo, Burkina Faso, Burundi, and Ethiopia. This shows that delayed breastfeeding initiation of a male child is a problem for these countries and requires concerted public health attention. Studies have shown that breastfed infants after an hour have a higher risk of mortality [11, 31, 35]. Given that three countries out of the six countries are in West Africa, child sex inequalities in EIBF might have been caused by some perceptions and socio-cultural practices and health-related factors which differs from countries to countries [36].

A study in West Africa found that male children and mothers from deprived households had a higher odds of delaying breastfeeding initiation [37]. The reason why countries in Central Africa and Southern Africa had lower odds of early breastfeeding inequalities could be because child gender preference are lower in those countries [38]. In the same vein, the current gap in EIBF could be the breastfeeding practice adopted in different regions and also gap in knowledge of the benefit of EIBF to a child irrespective of the sex [16, 37]. Similarly, these reasons could be attributed to child sex inequalities in breastfeeding initiation by mothers in Burundi. Although the specific reasons for the differences in the early initiation for male and female babies were unexplored by this study, breastfeeding counseling for women in these six countries may have to prioritize the promotion of EIBF for male children irrespective of the perceptions women have towards giving birth to a male child in these countries. The efforts to intensify breastfeeding initiation for mothers in these countries must be intensified to improve child health.

Despite this key finding concerning inequalities in EIBF in SSA, we found that mothers with one to three children recorded a higher prevalence of delayed initiation of breastfeeding. Mothers who lack experience of breastfeeding and its benefits would initiate breastfeeding late regardless of the sex of the child [39]. For example, Adhikari, Khanal [40], and Liben and Yesuf [39] found that mothers (in Nepal and Ethiopia) were more likely to breastfeed their children within an hour after giving birth as the birth order increases. Surprisingly, the study found a higher proportion of late breastfeeding initiation among mothers residing in urban areas in SSA. We expected that mothers in urban areas would have a lower proportion of late breastfeeding initiation compared to rural mothers. This is because urban mothers seem to have much access to antenatal care services and different information sources on breastfeeding [21, 26, 39].

### Policy and public health implications

The findings of the study have several implications for policy and public health. The results demonstrate the need to prioritize the promotion of EIBF not only for male newborns but also for females. In countries with significant differences between a child’s sex and breastfeeding initiation, health authorities may need to design specific interventions to encourage mothers to breastfeed both male and female children irrespective of the sex of their babies within the first hour of birth. Our findings support the WHO recommendation for mothers to breastfeed newborn babies within an hour [41]. Health education programs must be promoted to stop social norms and stereotypes that influence inequalities in EIBF of babies. Public health models that seek to reduce neonatal mortality risk must champion EIBF for both sexes. Health workers can help mothers to understand the consequences of early breastfeeding inequalities through continues promotion on education on benefits of early breastfeeding.

### Strengths and limitations

This study has several strengths and limitations. This is the first study to examine sex child inequality in EIBF in SSA to the best of our knowledge. The results will therefore, guide health authorities to promote early breastfeeding programs together with exclusive breastfeeding. Additionally, the study used data from a large nationally representative survey with relatively large sample size making it possible to generalize the study’s findings. Despite these strengths, there are also some limitations that need to be stated. The data’s cross-sectional nature do not allow causality, alsosince the breastfeeding initiation information was self-reported, there might be recall issues leading to underreporting or overreporting. Finally, the data pooled from different countries included in this study had DHSs conducted at different intervals between the years 2010 to 2020. As such, the results of those surveys conducted 10 years ago might not be applicable now.

## Conclusion

This study has unpacked the childhood inequality in EIBF in SSA. We found a higher odds for EIBF among women with females at birth in 24 sub-Saharan African countries. For country-level analysis, four countries, particularly Togo, Burkina Faso, Burundi, and Ethiopia, recorded a statistically significant association between child sex and breastfeeding. Despite this important finding, there was a higher prevalence of late breastfeeding initiation (More than 1 hour) among women aged 15–24, those who had their first child below the age of 20, women who were assisted by unskilled professionals during birth, residing in the urban, whose partner had no education, those who had home delivery, who had a delivery through cesarean section, those with no visit to ANC visit and women who were not exposed to media in SSA. To reduce inequality in EIBF among mothers, health education programs that promotes early child breastfeeding’s importance to pregnant women in SSA. Breastfeeding counselling must be promoted in Togo, Burkina Faso Burundi, and Ethiopia to encourage women to breastfeed their children early (within an hour). Since breastfeeding’s timely initiation improves child health and reduces infant deaths, we recommend that health professionals assist mothers to initiate breastfeeding early irrespective of the child’s sex.

## Abbreviations

SDG: Sustainable Development Goal
SSA: sub-Sahara Africa
DHS: Demographic and Health Surveys
COR: Crude Odds Ratio
AOR: Adjusted Odds Ratio
WHO: World Health Organization
EIBF: Early Initiation of Breastfeeding (EIBF)
LMICs: Low-and-Middle-Income Countries
HIV: Human Immunodeficiency Virus
AIDS: Acquired Immunodeficiency Syndrome
STROBE: Strengthening the Reporting of Observational Studies in Epidemiology
ANC: Antenatal Care
CI: Confidence Interval, and
SVY: Survey Stata Command
